# Effective governance for management of invasive alien plants: evidence from the perspective of forest and wildlife officers in Sri Lanka

**DOI:** 10.7717/peerj.8343

**Published:** 2020-01-06

**Authors:** E.M.B.P. Ekanayake, Yi Xie, Abubakar Sadiq Ibrahim, N.T.P. Karunaratne, Shahzad Ahmad

**Affiliations:** 1 School of Economics and Management, Beijing Forestry University, Beijing, China; 2 Department of Forest Conservation, Colombo, Sri Lanka; 3 School of Management and Economics, Beijing Institute of Technology, Beijing, China

**Keywords:** Biological invasions, Knowledge, Perception, Conservation officer

## Abstract

Invasive alien plants (IAPs) are a significant cause of socio-ecological change in Sri Lanka. Many studies have focused on the ecological dimensions of this problem, but few have addressed sociological factors such as the knowledge and perceptions of individuals and groups tasked with addressing IAPs. This study investigates how IAP issues are understood and perceived by professional forest and wildlife officers in Sri Lanka. The data analyzed were gathered using a questionnaire that covered three themes: the respondents’ ability to identify IAPs, the impacts of IAPs and the threats they pose, and knowledge regarding control and mitigation. The questionnaire was completed by 186 field officers, and the resulting descriptive statistics and a probit regression analysis were used to analyze the data. The results show that almost all of the participating forest and wildlife officers were aware of the problems associated with IAPs but more than 75% of them lacked an accurate understanding of scientific means for controlling them and control policies established by the government of Sri Lanka. Generally, wildlife officers had a better understanding than forest officers. In addition, the analysis shows that officers’ knowledge and perceptions of IAPs were positively correlated with their level of education and position within the organization. The analysis points to several recommendations for Sri Lankan officials when designing and implementing comprehensive policies and professional programs, particularly for lower-level field officers.

## Introduction

Invasive alien plants (IAPs) are a growing problem globally and recognized by the Millennium Ecosystem Assessment as the second largest biodiversity threat after direct habitat destruction (Latimer et al., 2004). Industries and sectors affected by IAPs include agriculture, animal husbandry, forestry, and human health (McNeely et al., 2001; Moran, Hoffmann & Zimmermann, 2005; Pejchar & Mooney, 2009; Marshall et al., 2011). One of the most remarkable characteristics of IAPs is their ability to dominate a wide range of habitats due to their vigorous growth and plentiful seed production. As IAPs spread, they alter the structure, composition, and functions of ecosystems, often leading to severe negative consequences (Perrings, Mooney & Williamson, 2010). Globally, trillions of dollars, amounting to approximately 5% of the world’s gross domestic product, are lost annually because of IAP impacts (Pimentel et al., 2001). However, not all of the IAP impacts are negative. For example, some invasive tree species have become important sources of timber and/or fuel and are commercially grown in forest plantations (De Wit, Crookes & Van Wilgen, 2001).

Tropical forests in Sri Lanka support a unique degree of biodiversity because of the area’s diverse topographical and climatic conditions. However, as in other developing countries, biological diversity in Sri Lanka is rapidly declining. Several studies in the past 10 years have found that a large number of Sri Lanka’s tropical forest species became extinct (e.g., *Impatiens repens*) in the preceding 400 years because of IAPs (Marambe et al., 2011; Ministry of Mahaweli Development and Environment (MMDE), 2015; Jayasuriya, 1995). Several earlier studies attempted to compile lists of IAPs in Sri Lanka, but the lack of generally accepted criteria to identify their invasive nature limited the studies’ value (Wijesundara, 2009; Bambaradeniya, Ekanayake & Gunawardena, 2001; Marambe, Amarasinghe & Gamage, 2003; Wijesundara, 2010). The Ministry of Mahaweli Development and the Environment (MMDE) conducted a risk assessment in 2015 and identified 20 plant species that were subsequently included in Sri Lanka’s national IAP list (Ministry of Mahaweli Development and Environment (MMDE), 2015). Most of the IAPs found in Sri Lanka were purposefully imported and introduced into the horticultural, agricultural, and forestry sectors and subsequently invaded natural landscapes, agricultural areas, and human settlements. As of 2010, nearly all terrestrial and inland aquatic ecosystems in Sri Lanka had been affected (Wijesundara, 2010). Given their numerous negative impacts on natural environments, IAPs have become a significant problem in Sri Lanka’s natural forests in general and in protected forest areas in particular (Marambe et al., 2011; Wijesundara, 2010; Bambaradeniya, 2002; Hitinayake et al., 2018).

Several government organizations in Sri Lanka have developed policy statements or working mechanisms to tackle issues related to IAPs. National-level policies directly dealing with IAPs are the National Wildlife Policy of 2000, National Environmental Policy of 2003, National Agriculture Policy of 2007, and National Policy on Invasive Alien Species of 2016. The National Policy on Invasive Alien Species (2016) provides the necessary legal structure to protect aquatic, marine, and terrestrial ecosystems, including agricultural and other man-made landscapes, and native biodiversity in Sri Lanka from risks associated with invasive alien species (Ministry of Mahaweli Development & Environment, 2016). Though several legislative actions have addressed the IAP problem in Sri Lanka, the laws have failed to address the problem of poor implementation of control policies and procedures (Ministry of Sustainable Development, Wildlife and Regional Development, 2018). Key stakeholder organizations such as Sri Lanka’s Department of Forest Conservation (DFC) and Department of Wildlife Conservation (DWC) so far have failed to create appropriate strategies for managing IAPs, especially in terms of efforts to eradicate them (Marambe et al., 2011).

The scientific literature related to IAPs has focused predominantly on their ecological impacts and on measures for controlling them (McNeely et al., 2001; García-Llorente et al., 2011). While some studies have highlighted the economic cost of IAPs (Moran, Hoffmann & Zimmermann, 2005; Wilgen et al., 2004), comparatively few have addressed the social dimensions of the problem, including considering the knowledge of practitioners (Estévez et al., 2014; Gaertner et al., 2016; Matthies, 2016). Many stakeholders are involved in IAP-related activities, including policymakers, scientists, commercial sectors, journalists, environmental officers, governmental and non-governmental organizations, and local communities (Lodge & Shrader-Frechette, 2003; Vanderhoeven et al., 2011). How well each stakeholder group understands the problems associated with IAPs and ways to mitigate those problems and their perceptions of the need for management play key roles in control and prevention of IAPs (Shackleton et al., 2019). IAPs are a consequence of human decisions and behaviors. Therefore, focusing on human belief systems and the behaviors that follow might be a more-effective long-term strategy for IAP management than focusing only on ecological factors (Vanderhoeven et al., 2011). The abilities and understanding of conservation workers will affect both the effectiveness and the efficiency of their actions (Hulme, 2007). Therefore, researchers and policymakers are increasingly recognizing the need to consider the social aspects of IAP management, particularly among professions that routinely confront IAP issues as part of their daily work (Stokes et al., 2006; Novoa et al., 2017).

Sri Lanka’s ecosystems are highly diverse, ranging from rainforests to savannas, and about 28% of the country’s plants are endemic (Gunatilleke, Pethiyagoda & Gunatilleke, 2008). Nearly all of Sri Lanka’s forest lands are state-owned and government-managed under the authority of the DFC and DWC. Biodiversity conservation and sustainable management of forests and forest resources are the main duties of DFC and DWC officers (Gunatilleke, Pethiyagoda & Gunatilleke, 2008; Ekanayake & Murindahabi, 2017). Therefore, the officers play a significant role in preventing the introduction and spread of IAPs in natural forests in Sri Lanka.

Effective and sustainable means of control of IAPs from natural forests cannot be fully accomplished without understanding stakeholders’ knowledge about and perceptions regarding controlling IAPs, and officers of the DFC and DWC are particularly important since they are the “boots on the ground” in efforts to eradicate the such species in forests. About 86% of the worlds’ forests are formally owned by governments (Agrawal, Chhatre & Hardin, 2008) and waged government officers were presumed to be responsible for protecting and managing the forests. However, very little is known about these officers’ knowledge and perceptions of IAPs. Therefore, evaluating how related individuals and groups understand the IAP problem is a necessity for implementing prevention and control programs in those sectors. To that end, we aimed to gain an understanding of how IAP issues are understood and perceived by two important groups of environmental officers in Sri Lanka.

## Methodology

### Conceptual framework

In this study, the term knowledge refers to the information, skills, and understanding individuals gain through learning and experience. Knowledge is based primarily on facts that define a problem and its potential solutions; it is derived from study and experiences. Perception has been defined as “the consciousness of particular material things present to sense” (Angell, 1906). As psychologists describe it, perception is, like sensation, something of an abstraction. Perception is related to an individual’s thoughts about a subject, and an individual’s perceptions are bound by the stimuli experienced by the individual and are affected by the individual’s mental contexts (Rock, 1985). In general, an individual’s knowledge cannot be fully elucidated without reference to the person’s perceptions (Snowdon, 1981). According to Schermerhorn, Hunt & Osborn (2000), individuals’ perceptions are processes in which they “select, organize, interpret, retrieve, and respond to the information from the world around them.” Therefore, perceptions can be influenced by a number of social-ecological factors (Shackleton et al., 2019). Thus, the concepts of knowledge and perception are closely linked because perceptions inform one’s knowledge of a subject (Strawson, 2008).

**Figure 1:**
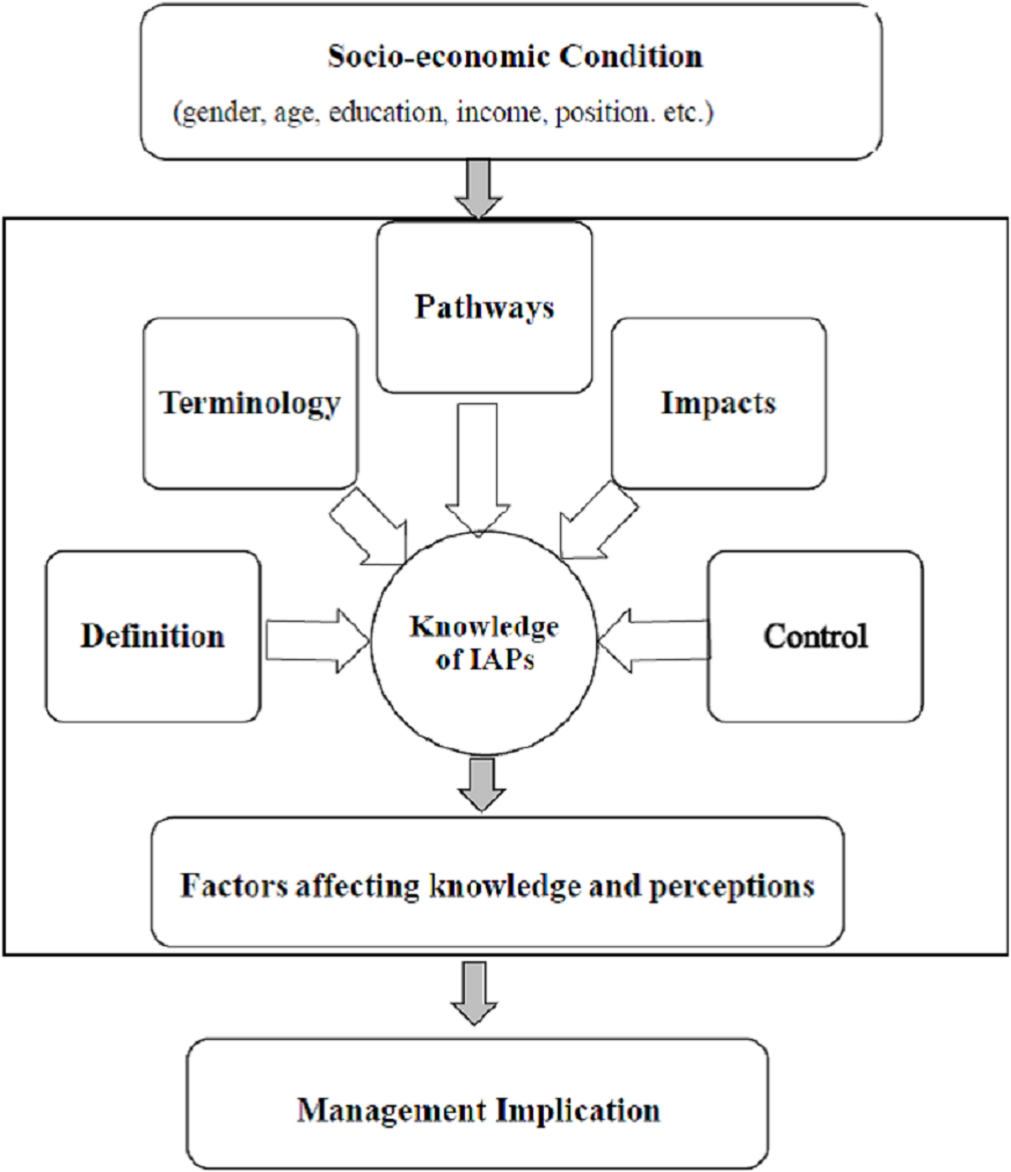
A conceptual framework of individuals’ knowledge and perceptions of IAPs.

To understand the two target groups’ knowledge and perceptions of IAPs, we developed a conceptual framework (Fig. 1) in which officers’ perceptions of control of IAPs result from mental processes at the individual level and are shaped by learning and experience (knowledge) regarding five sets of factors related to IAPs under three themes: (1) respondents’ ability to identify IAPs, (2) their knowledge of the impacts of IAPs and threats they pose, and (3) their preferences regarding control and mitigation actions. Making up the first theme are three factors related to IAPs: (1) the definition of an invasive plant, (2) terminology used, and (3) IAP spreading pathways. These represent the respondents’ ability to identify IAPs. Two factors make up the second and third theme: (4) the impacts of IAPs and (5) ways to control them. These five types of knowledge induce individuals’ perceptions regarding controlling IAPs. We evaluate respondents’ perceptions of IAP control using effective instruments such as legislation and policy.

Many socio-economic and demographic factors, including gender, age, level of education, income level, job position, and location of residence, can significantly affect the knowledge and perceptions of the DFC and DWC respondents (Mehta & Heinen, 2001; Bandara & Tisdell, 2003; Chowdhury et al., 2014). We incorporate these socio-economic factors into the model to identify the relationships between those factors and individuals’ knowledge and perceptions regarding IAPs.

### Study region and participants

Sri Lanka is a tropical island encompassing about 65,610 square kilometers of land area. The country lies between 5°52′ and 9°52′ latitude north and 79°41′ and 81°54′ longitude east. In 2017, the country’s population was recorded as 21.4 million people. According to the World Bank’s income classifications, Sri Lanka is a lower-middle-income country with a gross domestic product per capita of USD 4,073 in 2017 (World Bank, 2019). Sri Lankan society remains predominantly agrarian, but the country is moving toward a more urbanized economy focused on services and manufacturing (Athukorala et al., 2017). Its topography consists of three distinct peneplains: low country (altitude up to 300 m above mean sea level (MSL)), mid country (300–1,000 m above MSL) and up country (1,000–2,500 m MSL) (Ganashan, Baledira & Dassanayake, 1995), and the main determinants of the climate are rainfall and temperature. Average annual rainfall ranges from 1,250 mm in the dry zone to more than 5,000 mm in the wet zone. The average annual temperature is 27.5 °C (Panabokke, 1996).

According to data from a forest cover assessment conducted in 2010, 29.7% (about 1.95 million hectares) of Sri Lanka’s total land area is forested and managed by the DFC and the DWC (Edirisinghe, Ariyadasa & Chandani, 2012). In 2017, the DFC managed 17% of the total land area designated as forest reserves, proposed forest reserves, conservation forests, and national heritage and wilderness areas (Ekanayake & Murindahabi, 2017). The DWC managed approximately 8,500 square kilometers of forest area declared as wildlife protected areas and sanctuaries, accounting for 13% of the total land area of Sri Lanka (Ekanayake & Murindahabi, 2017). Protected areas in Sri Lanka account for 26.5% of total area. This is a greater percentage than in all of Asia and much of the world (World Resources Institute, 2006).

The questionnaire for collecting data for analysis was completed by both upper-level and lower-level staff members of the DFC and DWC in the root-level (lowest level) administrative units across Sri Lanka. Details regarding the participants in these root-level administrative units are shown in Table 1.

**Table 1:** Socio-demographic characteristics of the DFC and DWC participants (Ekanayake & Murindahabi, 2017).

Organization	Type of area controlled	Size of area controlled	Types of employees in root-level units	Roles of employees
Department of Forest Conservation	Conservation forest, Reserve Forest, Proposed forest reserves, National heritage and wilderness areas	12,708 km^2^	Range Forest Officers Beat Forest Officers Extension Officers Forest Field Assistant	Establishing and managing forest plantations. Monitoring and maintaining healthy natural forest cover. Thwarting offenses. Taking legal actions against offenders. Raising awareness of students and local communities.
Department Wildlife Conservation	National parks, Sanctuaries Strict nature reserves, Nature reserves, Forest corridors	8,500 km^2^	Wildlife Rangers Wildlife Guards Field Assistants	Daily monitoring of wildlife resources. Authentication of species’ health and protection. Thwarting offenses against wildlife. Taking legal action against offenders. Maintaining and managing habitats and food sources. Raising awareness of students and local communities.

In the DFC, Range Forest Offices are the main root-level administrative units that conduct field management activities. Sri Lanka is divided into 74 forest ranges, each managed by a Range Forest Office. Field activities include establishing and managing forest plantations, monitoring and maintaining healthy natural forest cover, thwarting offenses, and taking legal actions against offenders. The staff, consisting of beat forest officers, extension officers, and forest field assistants, is supervised by a Range Forest Officer.

Range Offices are the main root-level administrative units of the DWC as well and are divided into two types. Forest Park Offices (15) manage activities within national park boundaries while Range Offices (23) manage activities in wildlife sanctuaries and other protected areas. Both types of offices are supervised by a wildlife ranger and are staffed by wildlife guards and field assistants who conduct the field activities shown in Table 1.

Given their responsibilities, DFC and DWC staff members routinely confront problems associated with IAPs. When establishing forest plantations and reforesting harvested areas, for example, DFC officers have to control or eradicate IAPs that invade the newly planted sites to prevent them from causing significant damage to seedlings by competing for water, nutrition, and light. Climbing and vine-like IAPs also directly damage trees meant for timber production, preventing the desired tree species from filling in gaps, reducing growth of canopy trees, inhibiting forest succession, and promoting fires (Padmanaba & Corlett, 2014). Therefore, Range Forest Officers who manage forest plantations and reforestation projects must have a good understanding of how IAPs spread and ways to eradicate them.

Regarding the areas managed by DWC officers, the main threats posed by IAPs are severe reductions in appropriate wildlife habitat and food sources. Officers in Forest Park and Range Offices are frontline caretakers of wildlife living within sanctuaries. Therefore, the DWC staff must adequately understand all of the various factors that influence those ecosystems.

### Data collection and regression variables

We employed a systematic sampling method to select the sample offices and officers. There were 112 root-level administrative units when the survey was conducted. We first obtained 37 DFC offices (of 74) and 19 DWC offices (of 38) sampled by selecting every first office from the name lists released by the DFC and DWC. These offices were distributed evenly in terms of their geographic locations. Then, we selected every second officer from the name list of officers collected from each office and obtained four to eight individual officers from each office. Totally 200 individuals from the DFC offices and 150 individuals from the DWC offices were selected to participate in the survey and contacted the selected Range Forest Officers and Wildlife Rangers by telephone to request their participation in the survey. Next, we sent them the questionnaire by email and advised them to answer the questions without referring to any sources of information related to IAPs (e.g., books, the internet, colleagues) as we were interested in their existing personal knowledge and perceptions. Of the 350 recipients, 122 DFC staff members (61%) and 64 DWC staff members (43%) returned completed questionnaires, generating a dataset for 186 individuals for further investigation when data collection was completed in December 2018. Noticeably, all sample officers were invited to join in the survey voluntarily and anonymously without offending their privacy and generating ethic issues.

The questionnaire used to survey the officers was prepared in Sinhala, a national language in Sri Lanka, and contained both multiple-choice and open-ended questions grouped into three themes. The three themes addressed how the DWC and DFC identified such species, impacts the species have on forests and threats they pose, and control of IAPs and mitigation of their negative impacts.

We started our questionnaire using the definition of an IAP adopted by the Convention on Biological Diversity (CBD) to measure officers’ knowledge of what types of plants constitute IAPs (Secretariat to the Convention on Biological Diversity, 2009). In addition, we obtained information about the participants’ knowledge of the routes by which IAPs are introduced into new environments and pathways by which they spread (National Invasive Species Information Center of United State Department of Agriculture, 2019), including physical factors such as wind and general human activities (Convention on Biological Diversity (CBD), 2014). When formulating questions concerning the spreading pathways, we relied on Great Britain’s Non-native Species Information Portal, which listed 44 invasive pathways common to all taxonomic groups (Convention on Biological Diversity (CBD), 2014). We selected the ten most-significant pathways for the survey. Respondents answered questions regarding these pathways by ranking each as 1 for “yes/relevant” or 0 for “otherwise” (not relevant or no idea).

We next obtained information about the participants’ knowledge of the impacts of IAPs on forest lands in Sri Lanka and the long-term threats they pose to the environment and economy. During the study, respondents were asked to rank the seven negative impacts of IAPs by severity. A Wilcoxon signed rank test was used to investigate the significance of differences in the rankings of DFC and DWC employees.

We then surveyed the officers’ knowledge of control of IAPs and mitigation of their negative impacts in more detail. The questionnaire included questions related to the officers’ knowledge of policies for controlling and mitigating IAPs and on their perceptions of how well IAPs are being controlled on forest lands.

The questionnaire also collected information on five socio-demographic characteristics of the participants: gender, age, level of education, position (rank) in the department, and geographic area in which they worked. To identify potential links between those socio-demographic characteristics and individuals’ knowledge and perceptions of IAPs, we implemented a probit regression analysis. The probit regression system is well adapted to determining factors affecting participants’ responses to binary choices (Greene, 2010). We used participants’ socio-demographic characteristics as independent variables. Dependent variables related to their knowledge and perceptions were developed using seven questions representing the three themes of the study (see Table 2). Each question corresponded to an actual situation in officers’ work controlling IAPs in Sri Lanka. Questions 6 (knowledge) and 7 (subjective evaluation) related to IAP policies.

**Table 2:** Responses to question regarding the knowledge on definition and terminology of IAPs.

Questions	Response (Dummy; Yes = 1, No = 0)	Variable
**1. Definition:** Do you know the definition of IAPs?	Yes/No	*Define*
**2. Scientific name:** Do you know the scientific name of the IAPs in your forest area?	Yes/No	*Scientific*
**3. Origin:** Do you know the origin of the IAPs in your forest area?	Yes/No	*Origin*
**4. Skills:** Do you have knowledge and skill to understand the impacts of IAPs?	Yes/No	*Skills*
**5. Strategies:** Do you use any strategies to control IAPs?	Yes/No	*Strategies*
**6. Policy:** Do you have enough knowledge of IAP control policies?	Yes/No	*Policy*
**7. Satisfy:** Are you satisfied with your knowledge of IAP control policies?	Yes/No	*Satisfy*

In the analysis, gender was constructed as a dummy variable taking a value of 1 for men and 0 for women. The variable for participants’ rank/position was constructed as a dummy variable taking a value of 1 for all supervising DFC Range Forest Officers and DWC wildlife rangers and a value of 0 for all lower-level staff members. Similarly, education level was constructed as a dummy variable by dividing the participants into two groups: those with up to 13 years of school education and those with more than 13 years of school education. The work zone variable took a value of 1 for participants assigned to a wet ecological zone, 2 for participants assigned to an intermediate zone, and 3 for participants assigned to a dry zone. The occupation variable was formulated as a dummy variable in which DWC participants took a value of 0 and DFC participants took a value of 1.

## Results

### Socio-demographic characteristics of the DFC and DWC participants

Table 3 presents the socio-demographic characteristics of the DFC and DWC staff members who participated in the study. As shown in the table, 27% of DFC participants and 23% of DWC participants were supervising officers, and 73% of DFC participants and 77% of DWC participants were lower-ranking field staff members. The DFC participants’ average age was 41 years with a minimum of 27 and maximum of 59 and 78% were male. The average age of DWC participants was 35 years with a minimum of 26 and a maximum of 58 and 87% were male.

**Table 3:** Socio-demographic characteristics of the DFC and DWC participants.

Characteristic variable	Variable description	Percentage in DFC	Percentage in DWC	Percentage in total
Age	Respondent age (Mean value)	41	35	39
Sex	0 = female	22%	13%	19%
	1 = male	78%	87%	81%
Education	0 = school education ≤13 years	85%	78%	83%
	1 = school education >13 years	15%	22%	17%
Position	0 = lower ranks	73%	77%	74%
	1 = supervising officer	27%	23%	26%
Occupation	0 = wildlife officer	0%	100%	34%
	1 = forest officer	100%	0%	66%
Work location (Scale 1, 2, 3)	1 = wet zone	35%	19%	30%
	2 = intermediate zone	44%	27%	38%
	3 = dry zone	21%	55%	32%

Large percentages of the DFC (85%) and DWC (78%) participants reported having 13 years of education or less. Regarding their work locations, 35% of the DFC participants worked in a wet zone, 44% in an intermediate zone, and only 21% in a dry zone while 55% of the DWC participants worked in a dry zone. Though the protected areas managed by DWC cover all of the ecological regions in Sri Lanka, the majority of the national parks, wildlife corridors, and sanctuaries are located in dry zones. Thus, our sample of participants consists of a large percentage of DWC workers assigned to dry zones.

### Knowledge to the definition and terminology of IAPs

The surveyed individuals from DFC and DWC routinely perform field conservation work and so are aware of IAPs. Hence, 98% of DFC officers and 91% of DWC rangers were able to identify at least one species of IAP. Only a relatively small number of participants were well-versed in how IAPs are defined and in recognizing them (Table 4).

**Table 4:** Responses to questions regarding knowledge of definition and terminology of IAPs.

Question	Percentage in DFC		Percentage in DWC	
	Yes	No	Yes	No
1. Knowledge of CBD’s definition of IAPs	33%	67%	80%	20%
2. Knowledge of characteristics that identify at least one IAP of the twenty in the national IAP list	98%	2%	91%	9%
3. Knowledge of scientific names of IAP species	23%	77%	36%	64%
4. Knowledge on geographical origin of IAP species	10%	90%	20%	80%

Among the DFC participants, only 33% were familiar with the CBD’s definition of IAPs (Table 4). They tended to view IAPs as fast-growing plants that harm the environment but failed to recognize specific terms in the official definition. A majority of the respondents were not aware of the exotic (non-native) nature of the species. To investigate their knowledge of specific IAPs, the questionnaire asked respondents to list some invasive plants. The average number of species listed was four (minimum zero, maximum eight), and more than 50% of the respondents listed the same four plants: *Lantana camara, Mimosa pigra, Panicum maximum*, and *Clusia rosea*. Moreover, 94 DFC respondents (77%) stated that they did not know the scientific names of the species they recognized as IAPs and only 12 (10%) knew the geographic origin of the plants they mentioned (Table 4).

Department of Wildlife Conservation participants, on the other hand, were more knowledgeable about IAPs. Approximately 80% could define IAPs in line with the CBD definition, and the average number of IAP species listed was five (minimum two, maximum 13). More than 76% of the DWC respondents identified Sri Lanka’s six main terrestrial IAPs: *L. camara, Prosopis juliflora, P. maximum, Pteridium revolutum, M. pigra*, and *Ulex europeaus*. Their ability to identify the scientific names and geographic origins of the IAPs was similar to that of the DFC participants with 23 respondents (36%) who knew the scientific names and only 13 respondents (20%) who knew the geographic origin of the plants they mentioned.

### Knowledge of common spreading pathways

The one spreading pathway that was considered relevant by 100% of DFC and DWC participants was introduction of plants for ornamental purposes (see Table 5). The other top three pathways listed by DWC respondents were plants introduced for soil improvement (92%), introduced for agriculture or animal husbandry as food or fodder crops (87%), and accidentally introduced as contaminants of agricultural seed (81%). For DFC participants, the other three main factors were introduced for agriculture or animal husbandry as food or fodder crops (76%), introduced for soil improvement (73%), and introduced for forestry as plantation crops (64%).

**Table 5:** Responses to statements regarding the relevance of ten pathways by which IAPs spread.

Statement	Percentage in DFC		Percentage in DWC	
	Yes	Otherwise	Yes	Otherwise
1. Introduced for forestry as plantation crop	64%	36%	47%	53%
2. Introduced for agriculture or animal husbandry as food or fodder crop	76%	24%	87%	13%
3. Introduced for soil improvement	73%	27%	92%	8%
4. Introduced for ornamental purposes	100%	0%	100%	0%
5. Accidental introductions as contaminants of agriculture production (seeds)	20%	80%	81%	19%
6. Accidental introduction with timber trade	9%	91%	22%	78%
7. Accidental introduction with imports of used machinery, equipment and vehicles	11%	89%	16%	84%
8. Accidental introduction with imports of packaging materials and cargo	3%	97%	22%	78%
9. Accidental introduction with tourist industry	3%	97%	13	87%
10. Spreading via air currents	23%	77%	19%	81%

Overall, most of the accidental exposures were generally perceived as not relevant, and a majority of DFC (77%) and DWC (81%) participants were not aware that IAPs can spread into Sri Lanka on air currents.

### Knowledge to impacts of and threats posed by IAPs

For the DFC and DWC participants collectively, the largest share (44%) described IAPs as a problem only on state forest lands (Table 6). However, a sizable number (32%) listed their own properties, neighbors’ properties, and state forest lands as affected. Neighboring properties and state forest lands were listed by 16% of the participants, and their own properties and state forest lands were listed by 8% of the participants.

**Table 6:** Participants’ knowledge and perceptions of the severity of negative impacts of IAPs.

Statement	DFC		DWC	
	Ranking	Percent of respondents	Ranking	Percent of respondents
1. Reduce the productivity of lands	2	31	4	27
2. Reduce biodiversity	4	30	2	34
3. Lead to extinction of native species	5	57	5	55
4. Affect the structure and composition of ecosystems	1	57	3	47
5. Affect the wildlife habitat	3	29	1	50
6. Are harmful to human life	7	96	7	98
7. Lead to economic losses	6	87	6	64

Participants also ranked the severity of various threats posed by IAPs to provide data on their knowledge of the negative impacts of invasive species. Results of the Wilcoxon signed rank test showed no significant difference between the two groups. Nearly all of the DFC and DWC participants viewed IAPs’ negative impacts as a threat to natural environments. The results (Table 6) show that their knowledge is likely influenced by the department in which they work as half of DWC participants (50%) ranked effects on wildlife habitat as most severe while more than half of DFC participants (57%) ranked effects on the structure and composition of ecosystems as most severe. More than two-thirds of respondents in both groups ranked economic losses and harm to human life as least important.

Perhaps not surprisingly, among the DWC officers, 34% ranked reduced biodiversity second, nearly half (47%) ranked affecting the structure and composition of ecosystems third, and 27% ranked reducing land productivity fourth. Their ranking of extinction of native species as fifth was less intuitive. Among the DFC officers, approximately 31% ranked reducing land productivity second, 29% ranked effects on wildlife habitat third, 30% ranked reduced biodiversity fourth, and 57% ranked extinction of native species fifth.

### Knowledge and perceptions regarding control of IAPs and mitigation of their negative impacts

The survey data show that 62% of DFC participants and 72% of DWC participants had the knowledge necessary to manage IAPs. In terms of actively controlling the spread of invasive species, 42% of the DFC participants stated that they were actively working to control IAPs, 58% said they were not actively working on control, and 6% stated that they did not feel control was needed. When asked about whether control efforts were needed, 41% said IAPs needed to be controlled immediately and 53% said they needed to be controlled in the future.

Among the DFC participants who described themselves as actively working to control IAPs, 38% reported that they were monitoring and collecting information and 27% stated that they had included IAP control in the management plan that would be conducted in the next 5 years. A smaller number (21%) said that they had informed IAP experts about issues in their areas and anticipated receiving advice from those experts. All noted that they used no chemical control methods, which are prohibited in natural forests under the legal framework of the Forest Conservation Ordinance.

A greater percentage of DWC participants (66% vs. 42% of DFC participants) reported actively working to control IAPs with 43% indicating that they were removing the plants, 21% reporting that they were monitoring and collecting information about IAPs, 17% stating that they were including IAP control in the management plan that would be conducted in the next 5 years, and 4% reporting that they were anticipating receiving expert advice. The DWC respondents also reported using no chemical control methods, which are prohibited in their case by the Flora and Fauna Ordinance.

A majority of the DFC participants (63%) and nearly all of the DWC participants (94%) agreed that legislation should address IAP issues. Most DFC participants (81%) and less than half of DWC participants (31%) felt that future strategies to control IAPs should be designed by authorities at a higher level in the department. On the other hand, more than half of DWC officers (59%) and slightly fewer DFC officers (11%) preferred to have strategy decisions made by the root-level field officers. A relatively small number of DFC and DWC participants (8% and 10% respectively) preferred to have the national nature conservation authority take on that role. One of the more interesting results was that 92% of DFC participants and 75% of DWC participants disagreed with the statement that they were knowledgeable about Sri Lanka’s IAP control policies, indicating that they felt a need for more information on overarching policies affecting their work. A large majority of participants (72%) stated that community participation was important. Only 6% indicated that it was not important, and the remaining participants (22%) said they did not know.

### Factors affecting participants’ knowledge and perceptions

The results of the probit regression models are presented in Table 7. The models’ features indicate that these results are reliable. We tested for correlation between all explanatory variables and found that none of the correlation coefficients exceeded 0.5, which indicated that our explanatory variables could be applied into the model independently. We also used Cronbach’s alpha value to determine that our explanatory variables needed not be grouped into latent variable. All of the variance inflation factors were less than 2, indicating that our data did not suffer from multi-collinearity issues based on commonly used cut-off values (Billionnet, Sherrill & Annesi-Maesano, 2012; Hair et al., 2010).

**Table 7:** Results of factors affecting knowledge and perceptions of the respondents. Numbers in parentheses are standard errors.

	Define	Scientific	Origin	Skills	Strategies	Policy	Satisfy
Age	0.00 (0.02)	−0.01 (0.02)	−0.00 (0.02)	−0.00 (0.01)	−0.00 (0.01)	−0.02 (0.02)	0.00 (0.01)
Gender	0.32 (0.30)	−0.16 (0.29)	0.01 (0.43)	0.24 (0.25)	−0.26 (0.28)	1.89* (1.10)	0.04 (0.28)
Education	−0.37 (0.31)	0.75** (0.28)	0.55 (0.33)	−0.38 (0.25)	0.37 (0.27)	1.78*** (0.41)	−0.47 (0.38)
Position	1.43*** (0.27)	1.64*** (0.25)	2.32*** (0.39)	1.02*** (0.27)	0.76** (0.24)	3.00*** (0.69)	−0.91*** (0.31)
Working location	−0.06 (0.15)	−0.04 (0.15)	0.05 (0.22)	0.12 (0.13)	0.10 (0.15)	−0.11 (0.25)	−0.06 (0.15)
Occupation	−1.77*** (0.28)	−0.52* (0.26)	−0.92* (0.37)	−0.22 (0.23)	−0.00 (0.24)	−1.95*** (0.56)	1.00*** (0.30)
Constant	0.51 (0.69)	−0.36 (0.72)	−1.7* (1.04)	0.02 (0.62)	−1.09 (0.67)	−3.02** (1.45)	−1.33* (0.72)
Model features							
LR chi2	82.46	67.09	74.42	20.81	15.96	94.20	32.75
Prob > chi2	0.0000	0.0000	0.0000	0.0020	0.014	0.0000	0.0000
Pseudo R2	0.322	0.3070	0.4722	0.0869	0.0873	0.6259	0.1574
VIF	1.31	1.29	1.33	1.17	1.16	1.37	1.18
Number of observations	186	186	186	186	186	186	186

**Notes:**

* Statistically significant at *P* < 0.10.

** Statistically significant at *P* < 0.05.

*** Statistically significant at *P* < 0.01.

Of the three factors related to officers’ demographic characteristics, we found that age did not have a significant impact on their knowledge and perceptions, gender had a significant and positive impact only on officers’ perceptions of policies (*P* < 0.10), and education level had a significant and positive impact on officers’ knowledge of scientific names for IAPs (*P* < 0.05) and perceptions of *policy* (*P* < 0.01) variables.

In terms of working factors, we find a significant and positive correlation between participants’ positions (supervisor vs. lower-level) and their knowledge of IAPs (*define, scientific, origin, skills*, and *policy* at the *P* < 0.01 level and *strategies* at the *P* < 0.05 level). The exception is *satisfy*, for which there was significant and negative correlation (*P* < 0.01). These results indicate that upper-level supervisory participants were not satisfied with their knowledge of current IAP control policies.

Furthermore, the results show that participants’ occupation has a significant negative correlation with *define* (*P* < 0.01), *scientific* (*P* < 0.10), *origin* (*P* < 0.10), and *policy* (*P* < 0.01) with DWC participants more often feeling knowledgeable about identification of IAPs and relevant control policies than DFC participants. On the other hand, we find significant positive correlation between occupation and the *satisfy* variable (*P* < 0.01), indicating that DWC participants were less satisfied with their knowledge of current IAP policies than DFC participants.

## Discussion

Regarding effective management of IAPs, DFC, and DWC officers need to have a thorough knowledge of scientific aspects of IAPs. This knowledge is necessary for campaigns and activities aimed at raising awareness among young children, university students, and local community members (Ekanayake & Murindahabi, 2017). Sri Lanka’s National Wildlife Training Center and Forestry Institute have conducted long-term (earning diplomas or certificates) and short-term courses for root-level officers with the objective of providing them with necessary information and knowledge to embark on their careers as forest and wildlife officers. However, our findings reveal that the officers’ knowledge of scientific aspects of IAPs is limited. We find that they are limited by inadequate access to sources of scientific information (Wijesundara, 2010) and that there is likely a linguistic obstacle as well. Many of the participants in this study had completed their educations entirely in schools that used the national languages of Sri Lanka (Sinhala and Tamil). However, English is the primary language of scientific discourse, and that transfers of terminology from English to other languages have led to confusion (Richardson et al., 2000). Lagging curriculums could be part of the problem as well. In most developing countries, educational curriculums for forestry professionals do not reflect recent improvements in scientific knowledge related to biodiversity degradation (FAO, 2004). Additional education opportunities are needed so forest and wildlife officers can improve their knowledge of various terms used in invasion biology (Castri, Hansen & Debussche, 1990; Binggeli, 1994; Weber, 1997; Mack et al., 2000).

Out of six main IAP species identified by two target groups, *L. camara, M. pigra*, and *U. europeaus* have intensively invaded forest lands in Sri Lanka and have been identified as among “100 of the world’s worst invasive alien species” in the Global Invasive Species Database (Global Invasive Species Database, 2019). Foxcroft et al. (2013) reported that *L. camara, M. pigra, P. juliflora* and *U. europeaus* are invaders for 135 protected areas around the world. Moreover, *L. camara*, naturalized in 197 regions out of 843 region in world (Foxcroft et al., 2017). Our findings reveal that all respondents perceived introduction of plants for ornamental purposes as the main route of IAP invasion. This is consistent with the fact that Sri Lanka’s main IAP species are *L. camara, M. pigra*, and *U. europeaus*, which were introduced to Sri Lanka through the Royal Botanic Gardens as ornamental plants (Marambe, 2001). The majority (92%) of DWC participants agreed that plant introduction for soil improvement was the second main reason for plant invasions. This perception likely relates to the Victoria Randenigala Rantambe sanctuary, the country’s largest, which covers three ecological zones and has been invaded by *M. pigra*, which was introduced to control soil erosion on the banks of the Mahaweli River (Marambe, 2001). In addition, both groups listed introduction for agriculture or animal husbandry as a food or fodder crop as a top-ranked reason for biological invasion. This may be due to the fact that, of the six main terrestrial invasive species identified by respondents, a grass species (*P. maximum*) introduced to the country as a fodder crop, has invaded much of the island on both state and private lands (Wijesundara, 2010). In line with our study, prior findings by studies conducted in India and Singapore reported that IAPs had been introduced as potential ornamental or food species (Diwakar, 2003; Tan & Siang, 2003). Moreover, several studies conducted in temperate forests found that horticulture and the ornamental plant trade was the main spreading pathway of IAPs (Lambdon et al., 2008; Schmutz et al., 2007). On the other hand, studies conducted in the Maldive islands reported that the majority of biological invasions had taken place as a result of increasing international travel and trade (Marambe, Amarasinghe & Gamage, 2003).

Regarding their knowledge and perceptions of the impacts of IAPs, both the DFC and DWC participants were well aware of the threats posed by IAPs as they were visible in all of the ecological zones in the country. They identified the effects of IAPs on wildlife habitats and the structure and composition of ecosystems as most damaging. Practically, *L. camara, Chromolaena odorata*, *Myroxylon balsamum*, and *M. pigra* have invaded natural and semi-natural areas, leading to significant alterations in the composition and structure of natural forests (Marambe, 2001; Ranwala & Thushari, 2012), *P. juliflora* had invaded Bundala National Park (Gunarathne & Perera, 2016), and *L. camara* had invaded Udawalawe National Park, causing significant changes in the composition of native plants and habitats of wild animals (Wijesundara, 2009). In the past, the DWC has emphasized the need for eradication and control of non-palatable invasive plants that threatened wildlife in protected areas (Marambe, 2008). The other significant impact of IAPs from the perspective of DWC participants was reduction in biodiversity. IAPs have reduced biodiversity rather than enriched it (Van Wilgen & Richardson, 2012; Roy, Byrne & Pickering, 2012; Dickie et al., 2014). Corlett (2014) highlighted the growing threat from invasive species to biodiversity on tropical oceanic islands. A majority of the DFC participants in our study indicated that fast-growing invasive grasses were suppressing the growth of newly established seedlings in afforested and reforested sites, leading to poor productivity by the forest plantings. However, there is no empirical data generated through scientific research to support this argument. Researchers have much more work ahead to fill the many gaps in data regarding IAP issues in Sri Lanka. Consistent with a prior study of the limited direct impacts of IAPs on economies (Ranwala & Thushari, 2012), we found that DFC and DWC staff members did not recognize associated economic losses as an important threat posed by IAPs. Similarly, Shrestha et al. (2019) revealed that key stakeholder groups involved in managing the species lacked awareness of the long-term economic consequences posed by IAPs.

In terms of controlling IAPs and mitigating their impacts, DFC and DWC participants reported a lack of adequate knowledge about the country’s control policies, especially the National Policy on Invasive Alien Species (2016). This is likely related to their limited access to information about upper-level policies and regulations. Earlier studies (Bambaradeniya, 2002; Ministry of Sustainable Development, Wildlife and Regional Development, 2018) have found that the main barriers to managing invasive species in Sri Lanka were conservation workers’ lack of knowledge regarding the legal framework under which they operated and lack of proper coordination of the various stakeholder institutions that were directly or indirectly involved in making decisions. Therefore, governmental efforts to improve their knowledge of applicable policies and disseminate the regulations are badly needed (Wickramasinghe & Senaratne, 2010; Hulme et al., 2018). Participants in this study also emphasized the important role of local communities as a historical strength of their efforts (Crowley, Hinchliffe & McDonald, 2017). More negotiation and dialogue are needed to promote community participation in controlling IAPs.

The regression analysis in this study revealed that DWC participants were better at identifying scientific aspects (definition, scientific names, and geographic origins) of IAPs and were more knowledgeable about IAP control policies than DFC participants. On the other hand, all lower-ranked staff members in the DFC and DWC indicated that they lacked a comprehensive of all aspects of IAPs (definition, terminology, geographic origin, skills, management strategies, and policies). This may be due to the fact that lower-level staff members’ participation in decision-making processes in institutional and legal frameworks is quite limited. They naturally have little knowledge of IAP policies and how effective they have been. However, our analysis indicates that conservationists’ knowledge of scientific aspects and IAP control policies increases with education. For example, many DWC staff members had more years of education on average than DFC staff members and were more knowledgeable about IAPs than the DFC staff members (see Table 1). Obtaining additional education has frequently been considered an important step in improving individuals’ knowledge regarding biological invasive species (Courchamp, Chapuis & Pascal, 2003; D’Antonio et al., 2004; Pyšek & Richardson, 2010). We also find that many of the upper-level staff members (Range Forest Officers and wildlife rangers) are not satisfied with their knowledge of current IAP control policies. This appears to be due to a lack of collaboration and coordination between various institutions that hinders the flow of relevant information to DFC and DWC staff members.

Overall information gathered in the present study indicates that root-level staff members lack adequate knowledge of scientific aspects and legislative policies related to IAPs. To address this issue, local, regional, and international cooperation in forming information exchanges through workshops, poster campaigns, seminars, public lectures, and symposia is needed. Further, conservation authorities must support root-level conservation workers’ participation in regional and international events regarding IAPs. Planning for and adapting to biological invasions must be given more attention in Sri Lanka and other developing countries (Shrestha et al., 2019; Siwakoti & Shrestha, 2014). There is also a need for strong legislative policy measures to prevent intentional and unintentional introductions in Sri Lanka (Marambe, 2008).

The results of our regression analysis provide a sound basis for design and implementation of policies to address IAPs. Future studies could classify the officers into groups to capture more-detailed data and generate more-reliable results, allowing the various entities involved in controlling IAPs in Sri Lanka to improve the officers’ understanding of IAPs and enhance their capability to cope with invasions. Moreover, future studies could focus more on management practices and the governance activities of the officers.

## Conclusion

This study demonstrates that officers in forest and wildlife conservation departments in Sri Lanka generally have inadequate understandings and low knowledges of IAPs. Specifically, the officers lack knowledge of important scientific aspects (scientific names of the IAPs and their origin) of IAPs. The officers’ knowledge of pathways by which IAPs spread is also limited to only a few factors, such as introduction of plants for ornamental purposes, soil improvement, and agriculture and animal husbandry for fodder and food. Officers in the DWC, on average, are more knowledgeable than officers in the DFC regarding definitions of IAPs, terminology, and pathways by which they spread. Our regression results further support the conclusions drawn from the descriptive results. Correspondingly, DWC officers were less satisfied with current policies than DFC officers. Their positions in the departments had a significant impact on their knowledge and perceptions. Moreover, other demographic variables such as gender and education level had significant impacts in terms of specific kinds of knowledge and perceptions.

Inadequate understanding and low knowledge have negative impacts on the effectiveness of management of IAPs in Sri Lanka. We suggest that the officers’ capacity building, targeted to improving their understanding and knowledges of IAPs, needs to be in priority in strengthening IAPS control. More collaboration and coordination between stakeholders organizations are needed to promote dissemination of information about IAPs and that a comprehensive education program and training for staff members are needed to enhance their knowledge and perceptions. Community participation in implementing policies related to management of IAPs should be enhanced. Finally, we conclude that our findings can be help to understanding the magnitude and nature of IAP problems in particular ecosystems and can provide insights to promote effective management of IAPs.

## Supplemental Information

10.7717/peerj.8343/supp-1 Supplemental Information 1 Knowledge and perception data.

10.7717/peerj.8343/supp-2 Supplemental Information 2 Survey Questionnaire.

## Additional Information and Declarations

### Data Availability

The following information was supplied regarding data availability: The raw measurements are available as a Supplemental File.
