# Supplementary material for: Effective governance for management of invasive alien plants: evidence from the perspective of forest and wildlife officers in Sri Lanka
Source: PeerJ. 2020 Jan 6;8:e8343. doi: 10.7717/peerj.8343 (PMC6951289; doi:10.7717/peerj.8343)
Supplement: Supplemental Information 2 [file peerj-08-8343-s002.docx]

**SURVEY QUESTIONNAIRE**

**Effective governance of invasive alien plants:**

**A case study from perspectives of forest and wildlife officers in Sri Lanka**

**A short introduction:**

My name is Buddhika Ekanayake, a Doctoral student in School of Forest Economic and Management studies at **Beijing Forestry University, China.** This survey seeks to understand forest officer’s knowledge and perception towards the alien invasive Pants. Your contribution will help me to assess how concerned individuals and groups understand the alien invasive plant issue is an essential prerequisite for establishing programs and legislation within given sectors.

***Instruction:***

1. ***Write all the answers in Given space***
2. ***Write X for filling answer in Cube or blank***

**I.GENERAL INFORMATION ABOUT RESPONDENT**

1.1 Age (වයස)…

1.2 Gender (ස්ත්‍රී/ පුරුෂ): Male (පුරුෂ) Female (ස්ත්‍රී)

1.3Education qualification ( අධ්‍යාපන සුදුසුකම්):

a) Illiterate (පාසල් නොගිය) b) Primary (1-5 years)(ප්‍රාථමික )

c) Ordinary level (සා.පෙ)(1-10years) d) Advance Level (උ.පෙ) (1-13years)

e) University (උසස් අධ්‍යාපනය) (more than 13 years)

1.4 Occupation (රැකියාව): Level (Position) තනතුර …………

1.5Office address (කාර්යාල ලිපිනය):… ……………………………….

1.6 Monthly income (මාසික අදායම) ……………………………….

1.7 work experience (රාජකරි පලපුරුද)................................................

**II. General understanding of Alien invasive plant**

2.1 Do you know the words mean by **Alien invasive plant**? ඔබ දන්නවද ආගන්තුක ආක්‍රමණශීලි ශාක යන්නෙන් හදුන්වන්නේ මොනවද යන්න

Yes……….. No………

2.2. Do you have general knowledge of Alien invasive plant? ආගන්තුක ආක්‍රමණශීලි ශාක පිලිබඳ ඔබට දැනුමක් පවතිද Yes……….. No………

If yes, what do you know? පිළිතුර ඔව් නම්, ඒ සමන්ධව ඔබ දන්නේ කුමක්ද

2.3 How would you describe Alien invasive plant? ආගන්තුක ආක්‍රමණශීලිශාක යන්න ඔබ නිර්වචනය කරන්නේ කෙසේද.................................................................

2.4 What are their characteristics of Alien invasive plant? ඔබ දන්නා පරිදි, ආගන්තුක ආක්‍රමණශීලිශාකයන්හි ලක්ෂණ මොනවාද -

2.5 According to your knowledge how many Alien invasive plant recorded in your forest area? ඔබේ දැනුමට අනුව කොපමණ ආගන්තුක ආක්‍රමණශීලිශාක ප්‍රමාණයක් ඔබේ වනාන්තර ප්‍රදේශයේ වාර්තා වෙනවද

2.6 Can you list out the names of Alien invasive plant that you know (give common Name)? එම ශාකයන්හි සාමාන්‍ය ව්‍යවහාර නම පහතින් දක්වන්න

2.7 Do you know the scientific name of the species you mentioned in Question Number **3.6**ඔබ ප්‍රශ්න අංක 3.6න් දැක්වූ ශාක වල විද්‍යාත්මක නාමය දන්නවද?

Yes………….. No

If yes, (Mentioned how many you know) පිළිතුර ඔව් නම්, ඒවාහි නම් පහතින් දක්වන්න

2.8 According to your Knowledge, how these Alien invasive species are expansion or colonization in your forest area? (Please rank 1 to 10 according to the priority) ඔබගේ දැනීම අනුව මෙමආක්‍රමණශීලිශාකඔබේ වනාන්තර ප්‍රදේශයේ ව්‍යාප්ත වී ඇත්තේ කෙසේද ( ප්‍රමුඛතාවය අනුව 1 සිට 10 දක්වා පෙල ගසන්න)

- - Introduced for forestry as plantation crop( වන වගා ශාකයක් ලෙස හදුන්වාදී ඇත)
  - Introduced for agriculture /animal Husbandry as food or fodder crop (කෘෂිකර්මාන්තයේදී /සත්වපලනයේදී ආහාර ලෙස හදුන්වාදී ඇත)
  - Introduced for soil improvement ( පාංශු සංරක්ෂණය සඳහා)
  - Introduced for ornamental purposes ( අලංකාරය සඳහා )
  - Accidental introductionsas contaminants of agricultural, horticultural produce (seeds) කෘෂිකර්ම හා උද්‍යාන භෝග අමුද්‍රව්‍ය සමග මිශ්‍රව
  - Accidental introductionswith timber trade දැව වෙලදාමේදී
  - Accidental introductions with import of used machinery, equipment, vehiclesආනයනය කරන ලද උපකරණ හා යන්ත්‍ර සුත්‍ර සමග
  - Accidental introductions with import of packaging materials and cargo ආනයන කරන භාණ්ඩ ඇසුරුම් සමග
  - Accidental introductions with tourist industry – luggage, equipment සංචාරක කර්මාන්තයේ ප්‍රතිඑලයක් ලෙස
  - Spreading through air currents and water (sea) විදෙස්හි සිට සුළගින් හා සාගරය ඔස්සේ

2.9. Do you know the geographical origin of the Alien invasive species that you find in Forest?ඔබේ වනාන්තර ප්‍රදේශයේ වාර්තා වෙනආක්‍රමණශීලිශාකයන්හි නිජ භුමිය ඔබ දන්නවද Yes………….. No………….

2.10If yes, list out the geographical origin/ country of originපිළිතුර ඔව් නම්, එම ශාකයන්හි නිජ භුමිය දක්වන්න

**Iii. Effects and threats ofAlien invasive plant**

- 1. As a Sri Lankan, Do you think Alien invasive plants are problem? ශ්‍රී ලාංකිකයෙකු වශයෙන් , ඔබ සිතනවාද ආක්‍රමණශීලිශාකගැටළුවක් බව Yes No………
  2. As a professional, are you concerned about the problem of Alien invasive plant? ක්ෂේත්‍රයේ ප්‍රවීණයකු වශයෙන් ඔබ ආක්‍රමණශීලිශාක ගැටළුව පිළිබද සැලකිලිමත්ද Yes. No………
  3. In which context it makes problem to you (ආක්‍රමණශීලිශාක කුමන ආකාරයෙන් ඔබට ගැටළුවක් වී ඇතිද )

a) problem to my own property පුද්ගලික දේපලට

b) Problem to My Nabors property අසල්වැසි දේපලට

c) Problem to State land රජයේ දේපලට

- 1. What are the Effects and threats associated with the Alien invasive plant and natural environment? (Please rank 1 to 8 according to the priority) ආක්‍රමණශීලි ශාක මගින් ස්වභාවික පරිසරයට සිදුකර ඇති ගැටළු හා තර්ජන මොනවද ( ප්‍රමුඛතාවය අනුව 1 සිට 8 දක්වා පෙල ගසන්න)
- Reduces productivity of lands (භුමියේ එලදායිතාවය අඩුවේ )
- Suppress or replace existing species ( දැනට පවතින විශේෂ බාලවීම හා ඉවත්වීම)
- Reduces biodiversity in natural ecosystems (ස්වභාවික පරිසරයේ ජිව විව්ධත්වය අඩුවීම)
- Extinction of native species( දේශීය විශේෂ වදවීම
- Affect the structure and composition of ecosystems (පරිසර පද්ධති ක්‍රියාකාරිත්වයට බලපෑම් ඇති කිරීම
- Interfere with the wild life habitat (වන ජීවී වාසස්ථාන වලට බලපෑම් ඇති කිරීම
- Harmful to human life ( මිනිස් ජිවිත වලට හානිදායකවීම)
- Involves heavy costs to control (Economic loss) ( මර්ධනය සදහා අධික වියදමක් දෑරිමට සිදුවීම)

**iV.Control and solutions**

4.1 Do you have knowledge on Alien invasive plant control policies? (ආක්‍රමණශීලිශාක මර්ධනය සදහා දැනට පවතින ප්‍රතිපත්ති පිළිබඳ ඔබ දැනුවත්ද)

4.2Are you satisfied with existing Alien invasive plant control policies? (ආක්‍රමණශීලිශාක මර්ධනය සදහා දැනට පවතින ප්‍රතිපත්ති පිළිබඳ ඔබ සැහිමට පත්වෙනවද)

4.3At this time, do you have strategies to control the spread of Alien invasive plant in your forest area?ඔබ වනාන්තර ප්‍රදේශයේ ව්‍යාප්තව ඇති ආක්‍රමණශීලිශාක මර්ධනය සදහා දැනට ඔබ විසින් යම් ක්‍රියාමාර්ගයක් අනුගමනය කරනවද Yes…x… No……….

5.3If no, what is your idea to control Alien invasive plant in your forest area? (පිළිතුර නැත නම් ,ආක්‍රමණශීලිශාක මර්ධනය සඳහා ඔබගේ අදහස කුමක්ද

a) Need immediate control (ඉතා ඉක්මනින පාලනය කලයුතුයි)

b) Need to control in future ඉදිරි අනාගතයේදී පාලනය කලයුතුයි

c) No Need to control පාලනය කිරීමට අවශ්‍ය නැත

d) No idea (අදහසක් නොමැත)

4.4 If yes what are the strategies you use to control the spread of Alien invasive plant in your forest area?පිළිතුර ඔව් නම් ආක්‍රමණශීලිශාක මර්ධනය සදහා දැනට ඔබ විසින් අනුගමනය කරන ක්‍රියාමාර්ගය කුමක්ද ?

Collect Information (තොරතුරු එක්රැස් කිරීම

Monitoring නිරීක්ෂණය කිරීම

In expectation of competent advices (විශේෂාඥ උපදෙස් බලපොරොත්තුවිම)

To let the time work (ඉදිරි ක්‍රියාකාරී සැලැස්මට ඇතුලත් කිරීම)

Allow Ecological management ( ස්වභාවික පාලනයට ඉඩහැරීම )

Hoeing (පස් හෑරිම/උදුලු ගෑම

Pulling out (උදුරා ඉවත කිරීම)

Pulling out and fire destruction (උදුරා ගිනිතැබීම)

Herbicide (රසායනික ද්‍රව්‍ය යොදා මර්ධනය)

Eradication (තුරන් කිරීම)

Other (Specify) වෙනත් ……………..

4.5. Is these strategies are your own idea? මෙම මර්ධන ක්‍රියාදාමයන් ඔබගේ පුද්ගලික අදහසක්ද Yes………. No...

4.6. If not who make the strategies to control Alien invasive plant?පිළිතුර නැත නම් ,ආක්‍රමණශීලිශාක මර්ධනය සදහා ක්‍රමවේදයන් සකස් කරන්නේ කවුරුන්ද

a)Higher level officers of your department( ආයතනයේ ඉහල නිලධාරීන් )

b)Another nature conservation agency ( වෙනත් පරිසර සංරක්ෂණ ආයතන)

c) Other (Specify) වෙනත් ………………………………….

4.7Do you think the tools and strategies for Alien invasive plant control should come from a higher level of your department?ඔබ සිතනවද ,ආක්‍රමණශීලිශාක මර්ධනය සදහා ක්‍රමවේදයන් හා පිළිවෙත් ඔබ ආයතනයේ ඉහල ශ්‍රේණි මගින් ලබා දිය යුතු බව

Yes……. No………..

4.8 If yes/No, give the reason for your answer? ඔබගේ පිළිතුර සඳහා හේතුව පැහැදිලි කරන්න .. ....................................................................

4.9. As a solution to the Alien invasive plant problem, should there be a framework for environmental conservation professionals such as FD and WLD? ආක්‍රමණශීලිශාක ගැටලුව සඳහා විසදුමක් ලෙස පරිසර සංරක්ෂණ කාර්යයේ නියුතු ආයතන සතු යම් නිශ්චිත කාර්ය පටිපාටියක් තිබිය යුතු බව ඔබ සිතනවද Yes No……

4.10.Which framework are you willing to accept? (Please rank according to the priority) ඔබ යෝජනා කරන්නේ කුමන ආකාරයේ කාර්ය පටිපාටියක්ද (ප්‍රමුඛතාවය අනුව පෙල ගසන්න)

1. Gathering Information of the community about Alien invasive plant(ආක්‍රමණශීලිශාක පිළිබඳව ප්‍රජාවගෙන් තොරතුරු රැස්කිරම)
2. Limitation of use of exotic species (විදේශීය ශාක භාවිතය අඩු කිරීම)
3. Ecological trail testing before the introduction of any new exotic species (විදේශීය ශාක හදුන්වාදීමට පෙර පාරිසරික යෝග්‍යතාව පරික්ෂාව )
4. curb of commercialization of exotic species ( රට තුල විදේශීය ශාක වෙළදාම සිමා කිරීම )
5. Monitoring/eradication ( නිරීක්ෂණය හා තුරන් කිරීම)
6. Other (Specify) (වෙනත් …………………

v**. perception towards control**

- 1. Do you think, there should be government legislation to addressing the Alien invasive plant issue? ඔබ හිතනවද ,ආක්‍රමණශීලිශාක ගැටලුව සඳහා රාජ්‍ය මට්ටමේ ප්‍රතිපත්ති හා නීති පද්ධතියක් අවශ්‍ය බව

1. Strongly agree(ස්ථිරයි ( b) agree එකගයි (c) no Idea අදහසක් නෑ (d) disagree එකග නොවේ (d) strongly disagree (දැඩිව ප්‍රතික්ෂේපිතයි )
   1. Do you think it is necessary to raise public awareness in Alien invasive plant issue?ඔබ හිතනවද ,ආක්‍රමණශීලිශාක ගැටලුව සඳහා ප්‍රජාව දැනුවත්වීම අත්‍යවශ්‍ය බව

(a) Strongly agree(ස්ථිරයි ( b) agree එකගයි (c) no Idea අදහසක් නෑ (d) disagree එකග නොවේ (d) strongly disagree (දැඩිව ප්‍රතික්ෂේපිතයි )

5.3. Do you think that failure to control Alien invasive plant would threaten to nature and natural areas? ඔබ හිතනවද ,ආක්‍රමණශීලිශාක මර්ධනය කිරීමට නොහැකි වීම ස්වභාවික පරිසරයට තර්ජනයක් විය හැකි බව

(a) Strongly agree(ස්ථිරයි ( b) agree එකගයි (c) no Idea අදහසක් නෑ (d) disagree එකග නොවේ (d) strongly disagree (දැඩිව ප්‍රතික්ෂේපිතයි )

- 1. Do you think that failure to control Alien invasive plant would threaten to native flora? ඔබ හිතනවද ,ආක්‍රමණශීලිශාක මර්ධනය කිරීමට නොහැකි වීම දේශීය ශාක සඳහාතර්ජනයක් විය හැකි බව

(a) Strongly agree(ස්ථිරයි ( b) agree එකගයි (c) no Idea අදහසක් නෑ (d) disagree එකග නොවේ (d) strongly disagree (දැඩිව ප්‍රතික්ෂේපිතයි )

- 1. Do you think that failure to control Alien invasive plant would threaten to native fauna?ඔබ හිතනවද ,ආක්‍රමණශීලිශාක මර්ධනය කිරීමට නොහැකි වීම දේශීය සත්ව විශේෂ සඳහාතර්ජනයක් විය හැකි බව

(a) Strongly agree(ස්ථිරයි ( b) agree එකගයි (c) no Idea අදහසක් නෑ (d) disagree එකග නොවේ (d) strongly disagree (දැඩිව ප්‍රතික්ෂේපිතයි )

- 1. Do you think that failure to control Alien invasive plant would seriously destroy the recreational function of nature reserves?ඔබ හිතනවද ,ආක්‍රමණශීලිශාක මර්ධනය කිරීමට නොහැකි වීම පරිසරයේ අලංකාරය හා විනෝදාස්වාදය සඳහා යොගැනීමට දැඩි තර්ජනයක් විය හැකි බව

(a) Strongly agree(ස්ථිරයි ( b) agree එකගයි (c) no Idea අදහසක් නෑ (d) disagree එකග නොවේ (d) strongly disagree (දැඩිව ප්‍රතික්ෂේපිතයි )

- 1. Do you think that failure to control Alien invasive plan would seriously disrupt nature park visitation? ඔබ හිතනවද ,ආක්‍රමණශීලිශාක මර්ධනය කිරීමට නොහැකි වීම වන උද්‍යාන සංචාරක කර්මනත්යටදැඩි බලපෑමක් විය හැකි බව

(a) Strongly agree(ස්ථිරයි ( b) agree එකගයි (c) no Idea අදහසක් නෑ (d) disagree එකග නොවේ (d) strongly disagree (දැඩිව ප්‍රතික්ෂේපිතයි )

- 1. Do you think that failure to control Alien invasive plan would increase human animal conflict? ඔබ හිතනවද ,ආක්‍රමණශීලි ශාක මර්ධනය කිරීමට නොහැකි වීම මිනිසා හා සතුන් අතර ගැටුම් වැඩි වීමට හේතුවක් බව

(a) Strongly agree(ස්ථිරයි ( b) agree එකගයි (c) no Idea අදහසක් නෑ (d) disagree එකග නොවේ (d) strongly disagree (දැඩිව ප්‍රතික්ෂේපිතයි )
